# Does audit and feedback improve the adoption of recommended practices? Evidence from a longitudinal observational study of an emerging clinical network in Kenya

**DOI:** 10.1136/bmjgh-2017-000468

**Published:** 2017-10-23

**Authors:** Susan Gachau, Philip Ayieko, David Gathara, Paul Mwaniki, Morris Ogero, Samuel Akech, Michuki Maina, Ambrose Agweyu, Jacquie Oliwa, Thomas Julius, Lucas Malla, James Wafula, George Mbevi, Grace Irimu, Mike English

**Affiliations:** 1 Kenya Medical Research Institute (KEMRI), Wellcome Trust Research Programme, Nairobi, Kenya; 2 Nuffield Department of Medicine, University of Oxford, Oxford, UK; 3 Department of Paediatrics and Child Health, University of Nairobi, Nairobi, Kenya

**Keywords:** audit and feedback, quality improvement indicators, performance change.

## Abstract

**Background:**

Audit and feedback (A&F) is widely used in healthcare but there are few examples of how to deploy it at scale in low-income countries. Establishing the Clinical Information Network (CIN) in Kenya provided an opportunity to examine the effect of A&F delivered as part of a wider set of activities to promote paediatric guideline adherence.

**Methods:**

We analysed data collected from medical records on discharge for children aged 2–59 months from 14 Kenyan hospitals in the CIN. Hospitals joined CIN in phases and for each we analysed their initial 25 months of participation that occurred between December 2013 and March 2016. A total of 34 indicators of adherence to recommendations were selected for evaluation each classified by form of feedback (passive, active and none) and type of task (simple or difficult documentation and those requiring cognitive work). Performance change was explored graphically and using generalised linear mixed models with attention given to the effects of time and use of a standardised paediatric admission record (PAR) form.

**Results:**

Data from 60 214 admissions were eligible for analysis. Adherence to recommendations across hospitals significantly improved for 24/34 indicators. Improvements were not obviously related to nature of feedback, may be related to task type and were related to PAR use in the case of documentation indicators. There was, however, marked variability in adoption and adherence to recommended practices across sites and indicators. Hospital-specific factors, low baseline performance and specific contextual changes appeared to influence the magnitude of change in specific cases.

**Conclusion:**

Our observational data suggest some change in multiple indicators of adherence to recommendations (aspects of quality of care) can be achieved in low-resource hospitals using A&F and simple job aides in the context of a wider network approach.

**Key questions**

**What is already known about this topic?**

- Audit and feedback (A&F) interventions are on average only modestly effective in improving performance.
- Clinical networks may help support adoption of recommended practices and improvements in quality of care in high-income settings.

**What are the new findings?**

- Response to A&F may depend on (low) baseline indicator performance.
- Increasing degree of task complexity may be associated with more limited change in adoption of and adherence to practice recommendations in response to A&F.
- Standardised paediatric admission records linked to A&F were associated with improved documentation of clinical symptoms and signs at admission.
- Employing A&F in the context of a clinical network may offer a means to improve multiple indicators of adoption of and adherence to recommended forms of care in low-income countries.

**Recommendations for policy**

- Future work exploring responses to A&F will need to take account of the complex interplay between task type, intervention delivery, contexts and time.
- Clinical networks warrant further exploration as means to improve adoption of and adherence to recommended forms of care as part of improving quality of care in low-income countries.

## Introduction

Developments in information systems present an opportunity for low-income countries (LIC) to introduce routine audit and feedback (A&F) strategies for improving quality of care.^1–4^ While A&F is commonly used, systematic reviews of a large number of studies indicate considerable variability in effects that are on average only modest in improving performance.^5 6^ Questions therefore remain on how to optimise A&F^7^ while there is uncertainty on how to operationalise routine A&F, especially in LIC, including which behaviours it might influence, how it could be delivered or how many indicators may be the subject of feedback.

Recently, the Kenya Medical Research Institute/Wellcome Trust Research Programme, Kenya’s Ministry of Health, the Kenya Paediatric Association and University of Nairobi formed a partnership with 14 county-level (formerly district-level) hospitals in Kenya, referred to as the Clinical Information Network (CIN). The focus of CIN is to develop a coalition of partners focused on promoting adoption of recommended practices and using improved collection and use of hospital data as a central component. The CIN has been operational for over 2 years and aims to improve documentation of clinical findings on admission and to promote clinical classification of severity of illness, use of basic diagnostics and correct prescription practices as recommended in Kenyan guidelines^8^ (that are largely consistent with WHO recommendations).^9^

The theory and implementation strategy underpinning the formation of the CIN as a form of intervention has been described in detail elsewhere.^10 11^ In particular, the CIN provides external data-driven A&F as this is thought to help in overcoming health professionals’ limited abilities to accurately self-assess.^5 12^ It is hoped such feedback gives teams collectively the opportunity to evaluate their past performance, reflect and develop new actions and strategies to change and improve their practice facilitated by network engagement.^10 13 14^

Development of the CIN in Kenya provided the opportunity to describe responses to A&F delivered for multiple indicators reflecting different task types and with varying feedback intensities. Specifically, it offered us the opportunity to explore how responses might vary across facilities all engaged in a broader network intervention and explore the influence of a standardised paediatric admission record (PAR, a form of checklist) on documentation tasks. While all analyses are descriptive or exploratory they also provide a picture of changes across a broad set of indicators within a Kenyan clinical network.

## Methods

### Study sites and participants

The data were collected in 14 county hospitals with inpatient paediatric services. They were abstracted retrospectively by trained data clerks from routine case records after patients’ discharge. The population of interest was all children aged between 2 and 59 months admitted in these facilities with common serious childhood illnesses to whom national treatment guidelines apply, excluding surgical and burn cases.

### Data collection

A full description of the data collection process has been reported elsewhere.^15^ In brief, data were extracted from paper medical records by trained data clerks on the history of illness, physical examination, diagnosis, laboratory investigations, treatments and discharge plans.^15^ These data were entered into a REDCap (Research Electronic Data Capture) database.^15^ Standard operating procedures were used to train clerks and in written form to subsequently guide the abstraction process with regular supervision from the CIN team and hospital staff. Data validation and error checking was carried out at the respective sites before uploading the data on a daily basis to a central database. A second round of error checking was undertaken centrally and any corrections made in consultation with the clerks. For data quality assurance, a random sample of 10–15 case records in each hospital already entered by the clerks was selected independently by the CIN team, re-entered and cross-checked for accuracy and concordance every 3 months. Results were used to retrain clerks if needed.

### CIN activities

Participating facilities were selected purposefully with the Ministry of Health to represent two high-population regions with high and low malaria prevalence in Kenya.^16^ Hospitals were recruited into CIN in two phases. Nine hospitals were enrolled between September and October 2013 and had 25 months of CIN participation between December 2013 and December 2015. The remaining five hospitals were enrolled in February 2014 and had 25 months of CIN participation from March 2014 to March 2016.

In published work, we suggest the CIN as a form of intervention that employs 22 from a total of 73 discrete, identifiable implementation components (12 major and 10 minor components).^11 17^ The majority of these 22 components were related to three of nine conceptually coherent domains described by Waltz *et al* 18: ‘Use Evaluative and Iterative Strategies’, ‘Develop Stakeholder Interrelationships’ and ‘Train and Educate Stakeholders’ (for further information see ref 11). A&F is central to these wider implementation components and provides the basis for local evaluation and iterative reflection on progress. Utilising A&F results is a focus of the limited training that has been employed so that hospital teams understand the feedback reports and are better equipped to use them.^11^ In brief, CIN held two introductory meetings with a paediatrician, a nurse leading the paediatric ward team and a health records officer from each hospital participating in the same network event. Subsequent network meetings held 6 months brought a paediatrician (or their representative) together from each hospital. Audit reports were explained and key areas of active feedback discussed. Meetings including nurses and records officers occurred only annually to discuss progress and lessons learnt with the paediatricians regarded as key agents in any change process based on their role as mid-level (hybrid) clinical managers.^19^ Within CIN, hospitals are encouraged (but not financially supported) to implement standardised PAR forms previously associated with improved clinical documentation of admission events.^17 20 21^

### Indicators of adoption of or adherence to recommendations

We focus on adoption of or adherence to recommended practices guiding the admission care of hospitalised children specified in clinical guidelines. The indicators selected span three major domains at the point of admission including: (1) assessment and documentation of clinical signs and symptoms, (2) selecting (and documenting) investigation orders and adhering to syndromic classification of illnesses, and (3) appropriate drug prescribing. In the early phase of CIN, there was a focus on completeness of documentation for key clinical symptoms and signs as comprehensive data on admissions support wider value of the data. Disease-specific indicator development drew on earlier international efforts to define appropriate quality measures for paediatric hospital care in LIC hospitals,^22^ and prior experience in their evaluation.^20^ The indicators selected comprise measures of adherence to widely disseminated,^10^ national clinical practice guidelines, Kenya’s ‘Basic Paediatric Protocols’,^23^ and paid particular attention to malaria, pneumonia, diarrhoea, malnutrition and meningitis which are the most common causes of admission and death.^20^ We purposefully selected 34 indicators, from a total possible 74 indicators, that could be measured with the data available and linked to the specific Kenyan practice recommendations. We selected these 34 on the basis that they were linked to the five common serious diseases mentioned earlier, because sufficient data were present in every hospital over the study period (with the exception of malaria indicators linked to specific geographic areas) and because baseline performance was <90% (performance >90% at baseline provides little scope for improvement). A summary of indicator selection and classification is presented in online supplementary appendix figure 1a. Different indicators represented different tasks or behaviours and were therefore classified in a post hoc but preanalysis process into specific categories:

1. Simple documentation. Indicators representing a task that is conducted rapidly by the clinician based on a question or inspection and that can be documented typically as a Yes/No item in paper-based records including the standardised PAR form (similar to a checklist), for example, documentation of jaundice or cyanosis.
2. Difficult documentation. Indicators are of clinical assessment tasks that take slightly greater effort to conduct but for which documentation remains simple, for example, counting for 1 min and then recording the respiratory rate.
3. Cognitive work. Indicators require greater cognitive effort on the part of the clinician and we divide these into two classes: (A) those representing integration of knowledge on presenting clinical signs and symptoms in order to make a diagnosis, classify illness severity or select appropriate laboratory tests, for example, ordering a haemoglobin test after identifying severe pallor, or establishing a child’s HIV status; and (B) those related to following rules to prescribe recommended treatments (for some in correct dosages), for example, prescribing artesunate for severe malaria or calculating correct drug doses for penicillin when treating meningitis.

10.1136/bmjgh-2017-000468.supp1 Supplementary file 1

### Audit reports

Prespecified analytic scripts derived from explicit clinical logics linked to indicator definitions were developed and tested. Using the data in the central database, these analytic scripts were run to produce hospital-specific audit reports divided into general and disease-specific sections. A summary of the main report sections with examples of indicators that were subject to feedback is presented in online supplementary appendix table 2a.

Indicators predominantly reflected the proportion of patients to whom the indicator applied (eg, those with a specific disease) for whom the indicator criterion was achieved (eg, documentation of a key clinical sign or use of a recommended diagnostic test or treatment). Audit reports provided percentage compliance with the indicator and used a colour coding system to categorise performance: green for excellent (>90%); yellow for good (80%–90%); pink for fair (60%–79%); and red for poor performance (<60%).

Audit reports were prepared every 2–3 months and presented in tabular format. For each indicator, three performance measures were provided: first, representing a hospital’s most recent reporting period (2 months); second, a performance measure for that hospital for the entire period before the current 2 months’ reporting period; and third, a pooled measure of performance from all other hospitals. This reporting format was intended to provide hospitals with an indication of their progress and allow them to benchmark with the average performance of other hospitals. After the first 9 months, simple line charts of hospital-specific monthly performance measures were also provided focusing on indicators of clinical documentation and correctness of drug dosing. Feedback processes and a classification of feedback intensity are described in detail in box 1.

Box 1 **Feedback processes and categories of feedback intensity**

- The audit reports were provided to participating hospitals’ medical superintendents (equivalent to medical director), the team leaders in paediatric departments (the paediatrician(s) and nursing officer in charge) and the senior Health Records and Information Officer. Hospital-specific reports and soft copy presentations (MS PowerPoint slides) were circulated via email and printed copies were dispatched to hospitals. The reports most pertinent to the period preceding network meetings were reissued at this point and discussed. As Clinical Information Network (CIN) focuses on inpatient paediatric care, the paediatricians occupying a mid-level management role were a particular focus of feedback and were expected to pass on feedback results to their ward-based team.
- In the first 12 months of CIN activity, each hospital was also visited on two occasions by the clinical coordinator who delivered the feedback report in face-to-face meetings with hospital staff. On other occasions, the paediatricians were encouraged to deliver feedback to their teams in such fora using the supplied presentations. A senior paediatrician (the CIN clinical coordinator) also followed up the provision of reports with specific emails and phone calls (after 1–2 weeks) to the paediatricians highlighting specific areas of success and areas requiring continued improvement. These calls aimed to provide encouragement and support as part of building a sense of being within the network.

Depending on the nature of audit and feedback (A&F) provided, data are examined for indicators that could be classified into three groups:

- active feedback indicators—featured in written reports and subjects of discussion during workshops and interactions between the paediatricians and the clinical coordinator (either face-to-face, by email or telephone);
- passive feedback indicators—included in written reports but not the subject of discussion during workshops or by the clinical coordinator in phone calls or emails;
- no feedback indicators—indicators whose data were abstracted from medical records but that did not feature in any A&F reports provided to the hospitals (written or verbal) during the follow-up period.

### Statistical analysis

As hospitals joined the network at different times and our interest is in their response to network activities including feedback, we chose to analyse data from the first 25 months of a hospital’s engagement with the network. Time in these analyses cannot therefore be directly related to calendar dates. We focused on 34 indicators (listed in full in table 1). These included 12 simple documentation indicators (5 passive feedback and 7 no feedback), 8 difficult documentation indicators (2 active feedback, 4 passive feedback and 2 no feedback) and 14 indicators requiring cognitive effort spanning planning investigations, classifying disease severity in line with guidelines and correct prescribing.

**Table 1:** OR (95% CI) for the effect of time (month of follow-up) on individual indicators’ performance change

Type of task	Indicator	OR (95% CI) (performance change per month)	Correlation between random slopes and random intercepts
Simple documentation indicators			
Passive feedback	Central cyanosis	1.05 (1.00 to 1.09)	−0.89
	Pallor	1.03 (1.01 to 1.06)	−0.88
	Indrawing	1.09 (1.01, to 1.18)	−0.93
	Grunting	1.09 (1.03 to 1.18)	−0.93
	Acidotic breathing	1.11 (1.03 to 1.19)	−0.91
No feedback	Jaundice	1.06 (1.02 to 1.10)	−0.91
	Wheeze	1.06 (1.01 to 1.13)	−0.92
	Lymph nodes >1 cm	1.12 (1.03 to 1.20)	−0.90
	Wrist/rib signs for rickets	1.20 (1.06 to 1.27)	−0.92
	Thrush	1.13 (1.04 to 1.24)	−0.95
	Crackles	1.07 (1.02 to 1.12)	−0.90
	Stridor	1.13 (1.04 to 1.24)	−0.96
Difficult documentation indicators			
Active feedback	MUAC/WHZ	1.15 (1.08 to 1.23)	−0.80
	Respiratory rate	1.04 (1.01 to 1.09)	−0.79
Passive feedback	AVPU	1.06 (1.01 to 1.10)	−0.93
	Capillary refill	1.11 (1.05 to 1.16)	−0.88
	Skin pinch	1.09 (1.04 to 1.16)	−0.94
	Ability to drink	1.09 (1.03 to 1.17)	−0.95
No feedback	Pulse rate	1.02 (0.96 to 1.08)	−0.78
	Temperature	1.03 (0.99 to 1.08)	−0.88
Cognitive work indicators			
Group A: indicators requiring cognitive work to plan investigations or classify disease severity in line with guidelines			
Active feedback	HIV status known	1.19 (1.13 to 1.26)	−0.96
	LP result available	0.99 (0.96 to 1.03)	−0.75
	MPS results recorded	1.01 (0.98 to 1.03)	0.13
Passive feedback	Hb for pallor	1.04 (1.01 to 1.10)	−0.45
	Glucose for danger signs	1.02 (1.01 to1.12)	−0.74
No feedback	Non-classified malaria	0.91 (0.83 to 0.99)	−0.34
	Non-classified pneumonia	0.95 (0.92 to 0.98)	−0.67
	Non-classified dehydration	1.02 (0.99 to 1.04)	−0.55
	Non-classified diarrhoea	0.94 (0.86 to 1.03)	−0.65
Group B: indicators involving cognitive work to prescribe drugs in line with recommended guidelines			
Active feedback	Artesunate for malaria	1.17 (1.01 to 1.37)	−0.89
	Zinc for diarrhoea	1.01 (0.98 to 1.07)	−0.02
	Correct gentamicin dose	1.02 (0.99 to 1.04)	−0.79
No feedback	Ceftriaxone for meningitis	1.01 (0.97 to 1.05)	−0.61
	Elevated penicillin dose for meningitis	1.00 (0.98 to 1.04)	−0.69

AVPU, alert, verbal response, pain response, unresponsive; Hb, haemoglobin; LP, lumbar puncture; MPS, malaria parasite slide; MUAC/WHZ, mid-upper arm circumference/weight for height z-score.

### Outcomes of interest

In this study, the outcome of interest was indicator performance (ie, adoption of and adherence to recommended clinical practice) at patient level (patients were nested within hospitals). In 32 of 34 cases, the indicator measure was in binary form. Binary outcomes were also created for two composite indicators based on Kenyan national guidelines. First, penicillin dose for meningitis cases was calculated and transformed into a binary variable with penicillin dose greater than 80 000 IU/kg per dose representing the correct elevated penicillin dose for meningitis cases. Second, gentamicin dose per kilo body weight was calculated and transformed into a binary variable with >6 and <9 mg/kg representing correct gentamicin dosage.

To visually inspect performance over time and explore variability for individual indicators within the network, line plots of mean performance with 95% CIs adjusted for cluster size combined with scatter plots representing individual hospital’s monthly performance were used. For some indicators, plots were restricted to facilities with sufficient data during the entire follow-up period. For example, malaria is very uncommon in some sites and the indicator for artesunate use was not relevant in these settings. Colour-coded heat maps, green for excellent (>90%), yellow for good (80%–90%), pink for fair (60%–79%), and red for poor adherence to recommended clinical practice (<60%), were also used to contrast indicator-specific performance between the first 3 months and the last 3 months of participation in the network for all sites.

To characterise adoption and adherence to recommended clinical practice over time and quantify heterogeneity between hospitals we fitted a generalised linear mixed model with a binary outcome (logit link) and time treated as a continuous fixed effect. A likelihood ratio test statistic^24^ was used to test the most suitable random effect model (hospital random intercepts vs hospital random intercepts and slopes). To determine the appropriateness of using a linear or quadratic time effect we performed a likelihood ratio test. ORs and corresponding 95% CIs are used to measure the magnitude and direction of effects of time exposure to network activities on adoption and adherence to recommended paediatric practices. Results are presented for each indicator with respect to the nature of feedback to which they were subject. Estimated correlation coefficient between random effects (slopes and intercepts) was obtained for individual indicators.

We subsequently tested whether using the standard PAR had a generalised effect on recommended documentation practice over time. We restricted analysis to the 20 indicators the PAR provides the means to document (12 and 8 simple and difficult documentation indicators, respectively). Data on whether a PAR was used were recorded for each case at the point of data collection. For these analyses, follow-up time was categorised into three phases of CIN feedback: ‘1–8 months’, ‘9–18 months’ and ‘19–25 months’ on the basis of visual inspection of the plots of performance change that suggested early improvement in a number of indicators and to provide three approximately equal time periods. Interactive models with two categorical covariates were fitted for individual indicators. Significance of the interaction term between ‘time’ (moderator variable) and ‘PAR use’ (focal variable) was tested using likelihood ratio tests with ‘PAR not used’ and ‘1–8 months’ as reference groups for the variables ‘PAR’ and ‘time period’, respectively. The effects were measured as ORs with 95% CIs. Intracluster correlation coefficients (ICC) are used to indicate variation between hospitals in adoption and adherence to recommended clinical practice. All analyses were performed in R V.3.0.2. Alpha error was set at 0.05 for all statistical tests with no adjustment for multiple hypothesis testing.

## Results

### Descriptive

Between September 2013 and March 2016, a total of 78 239 admission case records were reviewed in 14 hospitals in the CIN network. Case records not within the 25 months of follow-up period for each hospital were excluded (n=14 849). Children less than 2 months old and children admitted as surgical or burn cases (n=3176) were excluded leaving 60 214 (76.9%) cases eligible for analysis (range 1397–7760 cases across hospitals reflecting varying workloads). Median admission age was 18 months (IQR, 8–41) with 26 660 (44.3%) of the eligible admitted children being female. Overall, 3707 (6.2%) deaths were recorded and the mortality rate varied significantly across the hospitals (range, 2.1%–8.5%).

**Figure 1:**
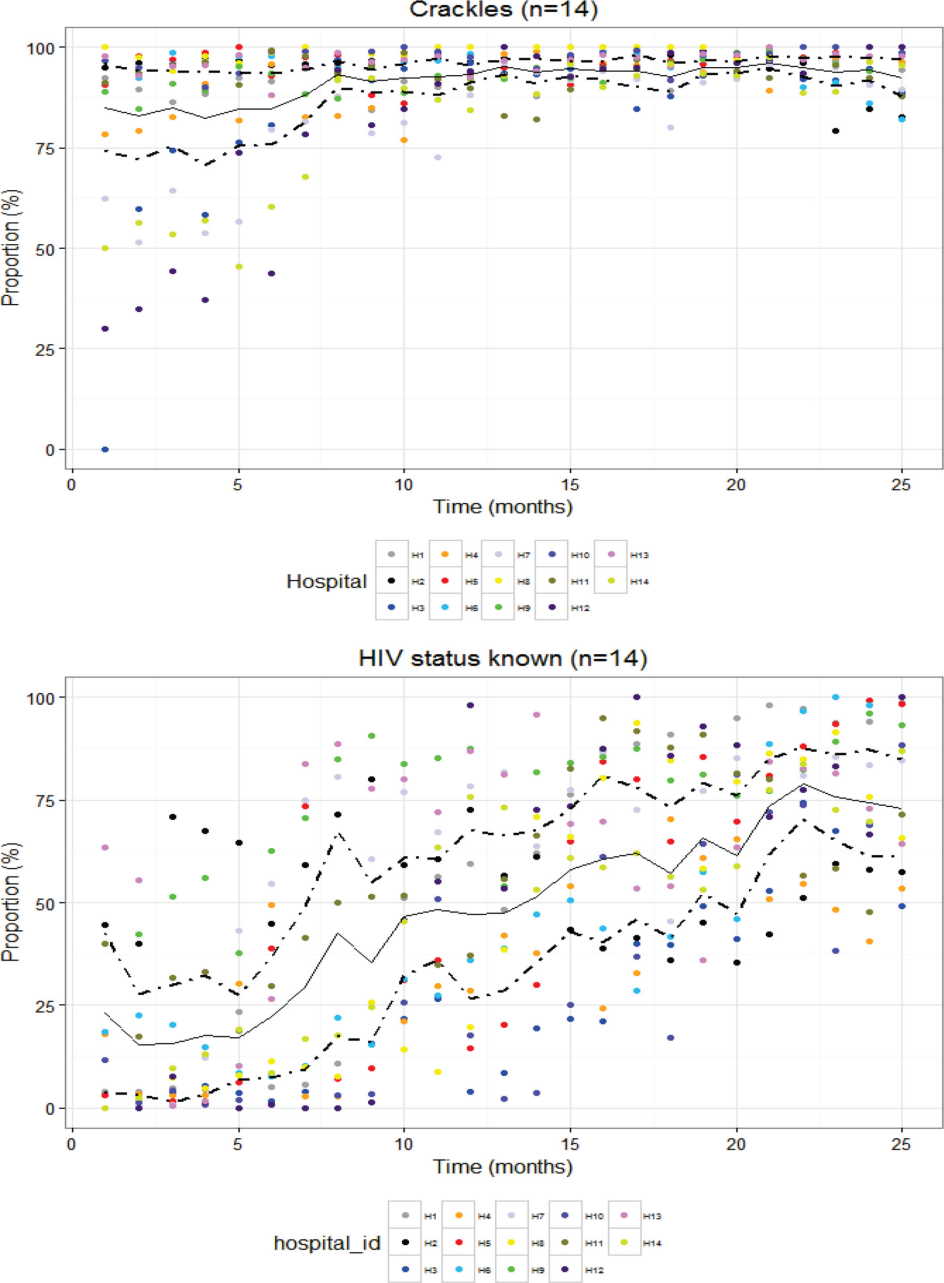
Cluster-adjusted mean performance (solid lines) at patient level with cluster-adjusted 95% CI (dotted lines) and individual measures of hospitals’ performance (dots) over the first 25 months of a hospital’s engagement with the network (time).

Examining the plots of changes in hospital-level performance over time, the nature of the task (whether simple documentation, difficult documentation or requiring cognitive work) and the form of feedback provided (active, passive and none) did not appear to have a major effect on changes in adoption and adherence to recommended practice (see online supplementary appendix 3a–e for plots for each indicator). Here we use two indicators to demonstrate some observed indicator trends over time (figure 1). This illustrates how relatively high baseline performance in adherence to recommended practice can leave little room for improvement (documenting crackles on auscultation) while lower and heterogeneous baseline performance was for some indicators followed by a rapid upward trend across hospitals over time (ascertaining HIV status).

**Figure 2:**
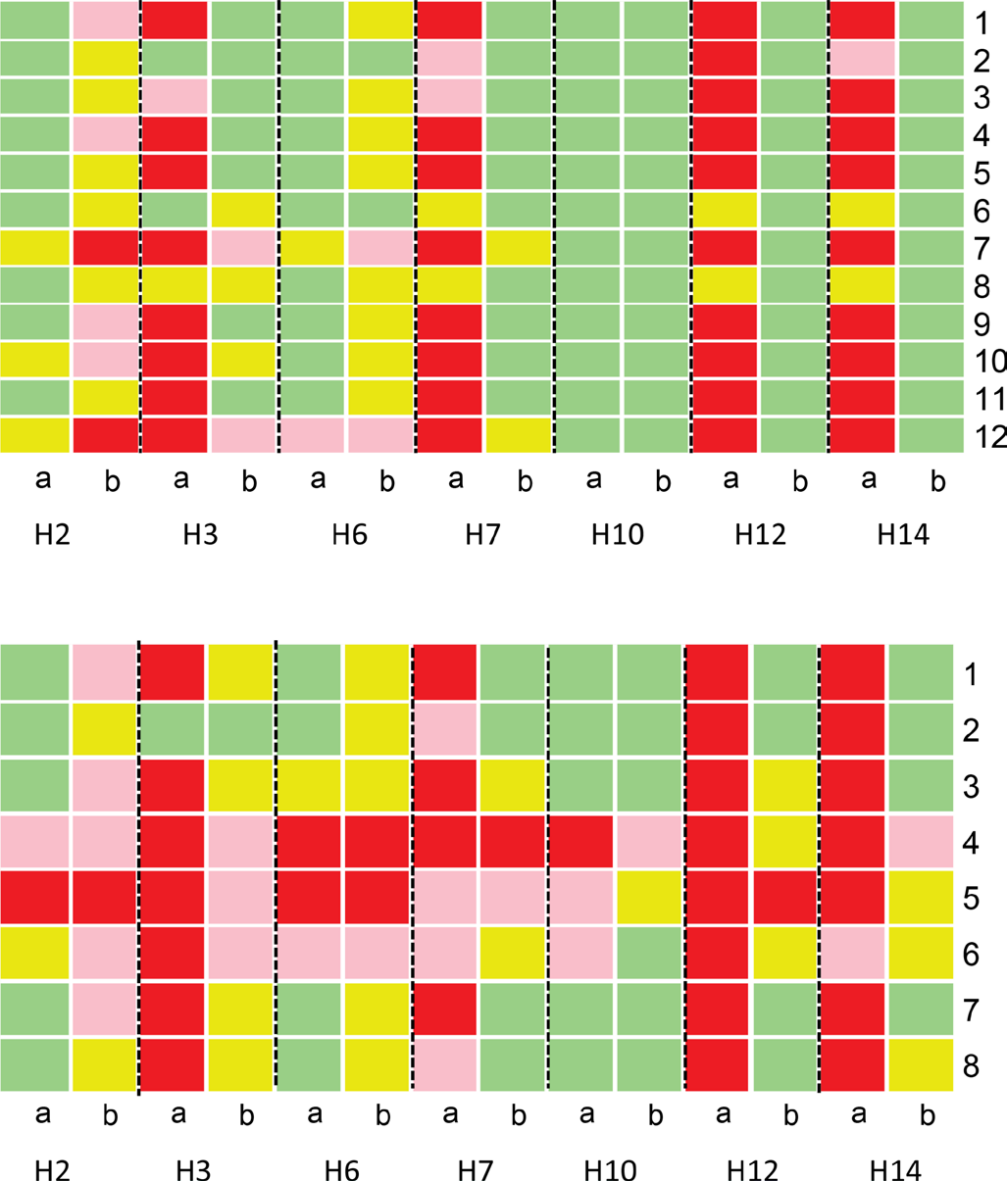
Comparison of documentation performance between the first 3 months (column a) and last 3 months (column b) of network participation for specific indicators (represented as number codes on the y-axis) requiring: simple documentation (upper panel) and difficult documentation (lower panel). Green shading represents excellent performance (>90%), yellow good (80%–90%), pink fair (60%–79%) and red poor (<60%). Upper panel indicator codes: 1: central cyanosis, 2: wheeze, 3: pallor, 4: jaundice, 5: indrawing, 6: acidotic breathing, 7: grunting, 8: thrush, 9: wrist signs for rickets, 10: crackles, 11: stridor, 12: lymph nodes. Lower panel indicator codes: 1: AVPU (alert, verbal response, pain response, unresponsive), 2: skin pinch, 3: can drink, 4: respiratory rate, 5: pulse rate, 6: MUAC/WHZ (mid-upper arm circumference/weight for height z-score), 7: temperature, 8: capillary refill.

**Figure 3:**
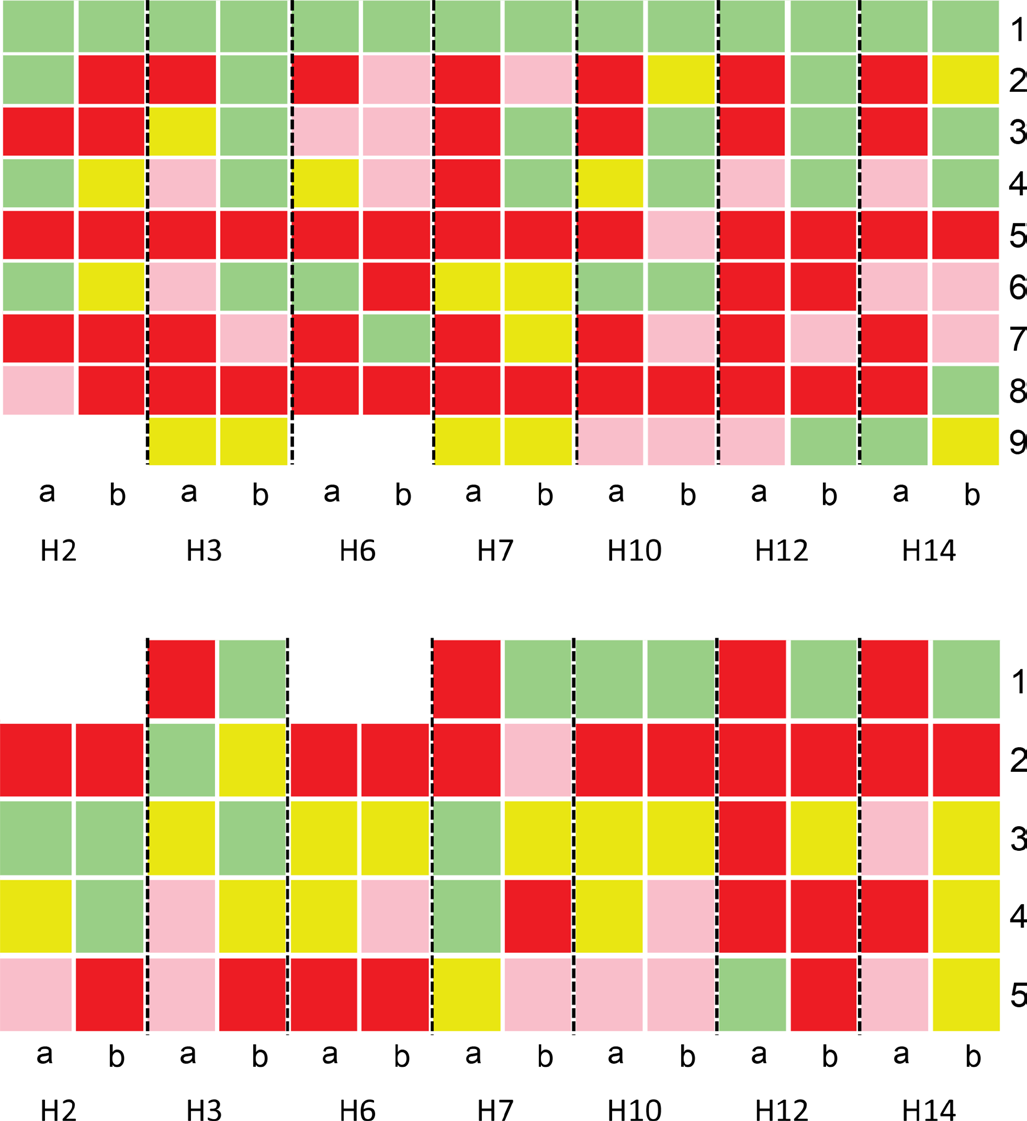
Comparison of documentation performance between first 3 months (column a) and last 3 months of network participation (column b) for indicators (represented as number codes on the y-axis) requiring cognitive work to: plan investigations or classify disease severity in line with guidelines (upper panel) and prescribe drugs in compliance with guidelines (lower panel). Upper panel indicator codes: 1: classified dehydration, 2: classified diarrhoea, 3: classified malaria, 4: classified pneumonia, 5: glucose for danger signs, 6: Hb (haemoglobin) for severe pallor, 7: HIV status known, 8: LP (lumbar puncture) results available, 9: MPS (malaria parasite slide) results recorded. Lower panel indicator codes: 1: artesunate for malaria cases, 2: ceftriaxone for meningitis cases, 3: correct gentamicin dose, 4: elevated penicillin dose for meningitis cases, 5: zinc for diarrhoea cases.

An assessment of indicator performance in the first 3 months and last 3 months of network participation revealed a number of findings of interest (see online supplementary appendix 4a–d for all indicators). Here for clarity we use seven hospitals to demonstrate some of these findings (figures 2 and 3). For simple and difficult documentation indicators, four hospitals had markedly worse performance, that is, poorer adherence to recommendations at baseline (H3, H7, H12 and H14) across indicator sets than others (figure 2). Then in four of seven hospitals there was clear improvement by the end of the period of A&F often for indicators with low performance at baseline. Of the remaining three hospitals, one was performing well at baseline and continued to do so, and the other two performed overall better at baseline. In general, difficult documentation indicators showed poorer performance at baseline (figure 2, lower panel) than simple documentation indicators (figure 2, upper panel) and more rarely reached high performance (green) at the end of 25 months. Two hospitals with very good performance at baseline demonstrated declines in a number of indicators over time (H2 and H6). For indicators requiring cognitive work on the part of clinicians (figure 3), adoption and adherence to recommended guidelines was more heterogeneous between hospitals and between indicators. For some indicators (eg, administration of glucose to children with danger signs and use of ceftriaxone for meningitis cases), low adherence to recommended clinical guidelines (colour-coded pink and red) often persisted across time (figure 3).

### The effect of time on indicator documentation practices

Although visual inspection of changes in indicator performance over time suggests greater improvement in the early months of A&F, likelihood ratio tests indicated that treating time (in months) as a quadratic effect had no clear advantage over treating it as a linear effect. As linear effects are easier to interpret, we therefore present these results for all 34 indicators classified by type of task and form of feedback provided in table 1. For all 12 simple documentation indicators (5 passive feedback and 7 no feedback), we found a significant positive time effect on adoption of recommended documentation practice (ORs, 95% CIs significantly greater than 1). The rate of improvement appeared indicator dependent with no apparent systematic difference between sets subject to passive or no feedback. For 6/8 difficult documentation indicators we found a significant improvement with time (table 1; 2 active feedback and 4 passive feedback). For 2/8 difficult documentation indicators subject to no feedback (pulse rate and temperature) there was no significant effect of time on adoption and adherence to recommended documentation practices. Out of nine indicators requiring clinicians’ cognitive work but not related to drug prescribing (group A), in five there was a significant positive change with time (one active feedback, two passive feedback and two no feedback indicators, table 1). In the remaining four indicators of group A requiring cognitive work, there was no apparent change in performance over time (two active feedback, two no feedback). Of five indicators requiring cognitive work related to drug prescribing (group B), there was a positive change over time in only one indicator (active feedback) while the remaining four (two active feedback, two no feedback) showed no general performance change over time (table 1).

Overall, indicators with low baseline performance and large baseline variance (eg, documenting examination for oral thrush and signs for rickets on wrist and ribs) had larger positive slopes (gain) in performance over time compared with indicators with higher baseline performance and low baseline variation (eg, pallor and jaundice). These results correspond with the patterns observed in individual plots where indicators with low baseline performance exhibited faster improvements (see online supplementary appendix 3a,c). For all indicators (table 1), a negative correlation between the random intercepts and the slopes suggests important hospital-specific effects, that is, hospitals with higher baseline performance evidenced slower rates of improvement than hospitals with lower baseline performance and vice versa.

### The effect of PAR use on simple and difficult documentation indicators over time

We found a significant positive effect of using a standard PAR on performance for all simple and difficult documentation indicators in the first phase of CIN feedback (1–8 months) (table 2).

**Table 2:** Relative ORs (95% CI) estimating the effect of PAR use and time on documentation of clinical sign indicator and symptoms

Indicator	Indicator performance at baseline (%) (first 3 months)	OR (95% CI) documentation period 2 (9–18 months)	OR (95% CI) documentation period 3 (19–25 months)	OR (95% CI) PAR used	OR (95% CI) PAR used Period 2 (9–18 months)	OR (95% CI) PAR used Period 3 (19–25 months)	ICC
Simple documentation indicators							
Passive feedback indicators							
Central cyanosis	88.1	0.90 (0.87 to 0.93)	0.95 (0.92 to 0.97)	1.06 (1.05 to 1.07)	1.05 (1.04 to 1.06)	1.04 (1.03 to 1.05)	0.16
Pallor	89.9	0.82 (0.78 to 0.86)	0.92 (0.89 to 0.96)	1.04 (1.03 to 1.05)	1.05 (1.04 to 1.06)	1.05 (1.04 to 1.07)	0.15
Indrawing	79.3	0.97 (0.94 to 1.01)	1.04 (1.02 to 1.06)	1.12 (1.11 to 1.13)	1.04 (1.02 to 1.06)	0.98 (0.94 to 1.01)	0.17
Grunting	76	1.04 (1.02 to 1.07)	1.09 (1.08 to 1.11)	1.14 (1.13 to 1.15)	0.99 (0.95 to 1.03)	0.85 (0.79 to 0.92)	0.18
Acidotic breathing	73.7	1.07 (1.05 to 1.09)	1.12 (1.10 to 1.13)	1.15 (1.14 to 1.16)	0.98 (0.92 to 1.02)	0.82 (0.74 to 0.89)	0.19
No feedback indicators							
Jaundice	92	0.86 (0.82 to 0.89)	0.93 (0.90 to 0.96)	1.03 (1.02 to 1.04)	1.05 (1.04 to 1.06)	1.03 (1.02 to 1.04)	0.15
Wheeze	83.7	0.96 (0.93 to 0.98)	1.00 (0.97 to 1.02)	1.09 (1.08 to 1.10)	1.04 (1.03 to 1.06)	1.03 (1.01 to 1.04)	0.16
Lymph nodes >1 cm	62.2	1.05 (1.01 to 1.08)	1.16 (1.13 to 1.18)	1.23 (1.22 to 1.25)	1.11 (1.08 to 1.14)	0.99 (0.93 to 1.04)	0.17
Wrist/rib signs for rickets	51.1	1.24 (1.19 to 1.27)	1.35 (1.34 to 1.36)	1.36 (1.35 to 1.38)	0.95 (0.85 to 1.05)	0.41 (0.33 to 0.50)	0.19
Thrush	60.9	1.15 (1.13 to 1.18)	1.22 (1.24 to 1.25)	1.24 (1.23 to 1.26)	1.01 (0.95 to 1.06)	0.68 (0.59 to 0.77)	0.18
Crackles	84.4	0.94 (0.91 to 0.97)	0.99 (0.96 to 1.01)	1.10 (1.09 to 1.11)	1.05 (1.04 to 1.07)	1.04 (1.02 to 1.05)	0.16
Stridor	66.8	1.10 (1.08 to 1.11)	1.16 (1.15 to 1.17)	1.17 (1.18 to 1.19)	1.07 (1.05 to 1.09)	0.72 (0.65 to 0.77)	0.19
Difficult documentation indicators							
Active feedback indicators							
MUAC/WHZ	26.7	1.64 (1.50 to 1.18)	1.83 (1.74 to 1.90)	1.81 (1.75 to 1.91)			0.18
Respiratory rate	70.3	0.89 (0.83 to 0.94)	0.82 (0.75 to 0.89)	1.14 (1.12 to 1.15)	1.16 (1.13 to 1.19)	1.21 (1.18 to 1.23)	0.17
Passive feedback indicators							
AVPU	87.4	0.95 (0.92 to 0.97)	0.97 (0.94 to 0.99)	1.07 (1.06 to 1.08)	1.05 (1.04 to 1.06)	1.05 (1.04 to 1.06)	0.16
Capillary refill	61.1	1.13 (1.08 to 1.17)	1.24 (1.22 to 1.26)	1.29 (1.28 to 1.30)	1.04 (0.97 to 1.10)	0.86 (0.77 to 0.93)	0.17
Skin pinch	71.2	1.04 (1.01 to 1.07)	1.13 (1.25 to 1.14)	1.18 (1.17 to 1.19)	1.04 (1.01 to 1.07)	0.87 (0.81 to 0.93)	0.17
Ability to drink	71.9	1.04 (1.01 to 1.06)	1.12 (1.11 to 1.14)	1.17 (1.16 to 1.18)	1.03 (0.98 to 1.06)	0.86 (0.79 to 0.92)	0.15
No feedback indicators							
Pulse rate	52.7	0.87 (0.78 to 0.97)	0.75 (0.64 to 0.86)	1.19 (1.15 to 1.23)	1.26 (1.18 to 1.34)	1.42 (1.33 to 1.49)	0.16
Temperature	81.5	0.95 (0.92 to 0.99)	0.89 (0.84 to 0.94)	1.11 (1.10 to 1.13)	1.07 (1.05 to 1.09)	1.10 (1.08 to 1.12)	0.16

AVPU, alert, verbal response, pain response, unresponsive; ICC, intracluster correlation coefficient; MUAC/WHZ, mid-upper arm circumference/weight for height z-score; PAR, paediatric admission record.

However, the impact of PAR use in the subsequent phase 2 (9–18 months) and phase 3 (19–25 months) of network participation was indicator specific, as in some cases there was insufficient room for further improvement (see online supplementary appendix 3a,c). A likelihood ratio test indicated significant interaction between time and use of PAR for all simple and difficult documentation indicators except MUAC/WHZ (mid-upper arm circumference/weight for height z-score) score. There were no apparent systematic differences in the effects of PAR use for indicators subject to active, passive or no feedback. There was a suggestion that hospital identity was moderately important in determining the level of adherence to recommended documentation guidelines across indicators after taking account of PAR use (ICC, range from 0.15 to 0.19).

## Discussion

This study sought to describe and explore responses to repeated rounds of A&F delivered to 14 facilities in Kenya of performance assessed using indicators representing adoption of or adherence to recommended practices articulated in Kenyan guidelines. The indicators reflect different task types and were subject to varying forms of feedback delivered as part of a network intervention over an initial period of 2 years. The potential influence of a specific PAR (a form of checklist) on a number of documentation tasks was also explored. A Cochrane review of 140 randomised trials of A&F interventions reported only modest improvement in indicators of quality care in half of the studies.^6^ There are however few included studies from LIC. The review authors and additional research suggest that variability in effectiveness of A&F interventions may depend on differences in context, feedback design and the task the indicator reflects.^5 13 25^ To our knowledge, there are no other quality improvement initiatives/studies going on in hospitals participating in CIN. In addition, CIN is the first study of its kind in an African LIC context that aims to use routine data linked to repeated A&F cycles to promote adoption of recommended paediatric practices. Of the 34 clinical performance indicators examined, we found significant improvements in adoption of and adherence to good clinical practice recommendations in 24/34 (70%) indicators and no indicator showed deteriorating average performance across the 14 hospitals over time though there were setting specific instances of falling performance. Classified by type of task, 12/12 simple documentation indicators, 6/8 difficult documentation indicators and 6/14 indicators of tasks requiring greater cognitive effort showed on average a significant positive change with time. Although our data are purely descriptive, they do suggest, in the aggregate, that any response to A&F and wider network activities may depend on (low) baseline indicator performance,^14^ and that increasing degree of task complexity may be associated with lower adoption and adherence to recommended clinical practices.^13 14^

Using a PAR, linked to A&F, was associated with improved documentation of clinical signs, both simple and difficult documentation indicators. The greatest effect was in the first 8 months of CIN A&F cycles and network activity after which many hospitals had attained high performance levels resulting in a ceiling effect.^25^ Our results support findings that standardised clinical records (checklists) may help support improvements in quality of the documented assessment in maternal and child care in LIC,^17 22 26^ but are contrary to findings from some studies in surgical or acute medical settings.^27 28^

We did not design a prospective study to test the effect of the intensity of feedback on indicator performance. We characterised indicators as being provided in an active or passive form as part of A&F cycles or not actually being the subject of feedback at all (see box 1 for definitions). For indicators examining documentation practices, our data suggest little influence of the intensity of feedback consistent with the findings of one study in the USA.^25^ This is likely related to the cross-cutting effect of implementing the structured PAR that prompts documentation of all indicator items in the context of wider network engagement. It is perhaps worth noting, however, that the two documentation indicators that were not subject to any feedback but are included on the PAR (recording temperature and heart rate measurement) showed no improvement at all. For indicators reflecting tasks requiring some cognitive work and based only on descriptive analyses, we could not discern any clear effect of the nature of feedback. However, for two indicators (prescribing of artesunate and ascertaining HIV status), considerable improvement was seen in the face of active feedback and low performance at baseline. In these two cases and in the case of documentation of nutritional status (MUAC or WHZ), major performance improvements may also have been influenced by the use of data to advocate for better supply of resources (MUAC tapes, artesunate and HIV test kits) at managerial levels.^16 29^

Although descriptive, our data demonstrate marked variability across facilities in adoption and adherence to recommended paediatric care guidelines at baseline and subsequently considerable variability in performance change across indicators, place and time. In a small number of hospitals for specific indicators with a high baseline performance even occasionally declined. This heterogeneity supports the fact that context is likely to be a powerful influence on broad improvement interventions relying on routine A&F and is likely to be attributable to organisational and contextual factors at mesolevel and microlevel,^30^ in keeping with the general feedback intervention theory,^31^ and our earlier findings in cross–sectional work.^32^ To promote change, and in keeping with Hysong’s rearticulation of feedback intervention theory,^31^ we aimed to provide timely, individualised (to hospitals), non-punitive and to a degree customisable feedback,^2^ to team leaders. However, such feedback was provided within the context of a much broader network intervention that aimed to engage hospitals and these team leaders in improving care.^10 33^ How each individual team leader acts to motivate team members to overcome behavioural and contextual barriers to compliance with guideline recommendations^34^ may be an important aspect of the context. Examining the effects of context and how this influences implementation success will be best informed by specific qualitative research.

There are calls for future work on A&F to test the effects of specific individual components of feedback, ideally in randomised controlled trials^14^; however, this may only be possible in carefully controlled studies where individuals or small facilities are the unit of randomisation. Such trials with a focus on internal validity may have limited external validity, especially for forms of feedback that are more typically or feasibly delivered to larger organisational units. Cluster randomised trials can be useful but may only be practical where there are already large, high-quality routine data systems spanning large numbers of facilities.^35^ It is also important to realise that A&F may most often be delivered as part of a broader package of change strategies such as in the CIN. Mixed methods and longitudinal studies on A&F and cointerventions may therefore be particularly useful in LIC where improving quality is now a major concern.

### Limitations of the study

Our study was limited in several ways; first, we are able to work with only 14 hospitals and this may have introduced selection bias. Further, the few study sites limited the power to perform a two-level hierarchical analysis which may have been useful in exploring hospital-level characteristics influencing performance change. Second, our data only allow us to conduct exploratory analysis and we were unable to collect performance data prior to the onset of network activities. There also remain important questions about how we should characterise the tasks that indicators represent. We took a pragmatic approach assigning indicators to a priori defined groups based on our clinical understanding of the type of work required to complete a task. Further conceptual and empirical work is likely to be important to this subject. Despite these limitations, our work demonstrates that it is possible to generate data during routine care and examine changes in care over time in response to intervention in an LIC.

## Conclusion

This observational study reports the effect of A&F delivered as part of a wider network strategy with, averaged across 14 hospitals, positive changes for 24/34 indicators of the adoption of or adherence to recommended practices for care delivered by junior clinicians when admitting children. For simple and difficult documentation indicators that were largely responsive to A&F and wider change efforts, the nature of feedback seemed less important than the overall effect of introducing a standardised clinical form to prompt improved adherence to recommended documentation practice in paediatric care. There was some suggestion that indicators measuring tasks that required greater cognitive effort were less likely to respond to A&F. Some of the most marked improvements were made in areas where baseline performance was low, where there was active feedback and where important contextual changes including developing local solutions to resource challenges occurred. Future work exploring how analysis of routine data to provide A&F can complement wider change efforts will need to take account of the complex interplay between task type, intervention delivery, contexts and time.
