# Correction: Does audit and feedback improve the adoption of recommended practices? Evidence from a longitudinal observational study of an emerging clinical network in Kenya

**DOI:** 10.1136/bmjgh-2017-000468corr1

**Published:** 2017-12-07

Gachau S, Ayieko P, Gathara D, *et al*. Does audit and feedback improve the adoption of recommended practices? Evidence from a longitudinal observational study of an emerging clinical network in Kenya. *BMJ Glob Health* 2017;2:e000468.

This article has been corrected since it was first published. The ninth author’s first name was misspelt during article submission. The correct spelling is Jacquie Oliwa.

