# Supplementary material for: Does audit and feedback improve the adoption of recommended practices? Evidence from a longitudinal observational study of an emerging clinical network in Kenya
Source: BMJ Glob Health. 2017 Oct 23;2(4):e000468. doi: 10.1136/bmjgh-2017-000468 (PMC5663259; doi:10.1136/bmjgh-2017-000468)
Supplement: Supplementary file 1 [file bmjgh-2017-000468supp001.pdf]

MATERIAL AND METHODS

DNA extraction

The concentration and purity of the DNA was assessed using a Nanodrop 2000 spectrophotometer (Thermo Scientific, Wilmington, DE, USA).

mtDNA copy number relative quantification

DNA samples were initially diluted to 2ng/µL from the stock DNA using DNA Tris-EDTA buffer solution. The DNA samples were further diluted for 0.4ng/µL. For each sample, two primer pairs were used to amplify the $tRNA^{Leu(UUR)}$ gene – mtDNA and *ß-2-microglobulin* (*ß2M*) gene - nuclear DNA, in separate wells. The primers sequence and qRT-PCR amplification procedure were described previously (1). A qRT-PCR amplification for each sample was performed in a final volume of 10 µL, using the 2× SYBR SuperMix (iTaq SYBR Green Supermix with ROX, BioRad). Primer concentration used in qRT-PCR was 5 µM. All samples were run in triplicate for both mitochondrial and nuclear genes on a 96-well plate with a 7500 Fast Real-time PCR system (qRTPCR; PE7500 real-time PCR instrument; Applied Biosystems, Foster City, CA, USA). A negative and a positive control were also included in each run, to control possible contaminations and to act as internal calibration. Standard deviations for the threshold cycle value were accepted at 0.50. Otherwise, the analysis was repeated. The results were analyzed with the 7500® v2.0.4 software (Applied Biosystems).

Statistical analysis

The statistical power and effect size of sample were estimated using the power analysis for ANOVA designs software (http://www.math.yorku.ca/SCS/Online/power/) (setting α=0.05, N≥50, with 5 levels of factor for power, Δ=1.0).

Normal distribution was assessed using the Kolmogorov-Smirnov test. A power transformation was performed to normalize mtDNA copy number values and to satisfy the homogeneity of variances' assumption for the errors. We performed power transformations and the only transformation that confirmed the homogeneity of variance between groups, as assessed by Levene's test for equality of variances (p=0.519), was the (1-1/x) transformation (2), which allowed us to perform parametric tests.

REFERENCES

1. Venegas V, Halberg MC. Measurement of mitochondrial DNA copy number. Methods Mol Biol. 2012;837:327-35.
2. Hoaglin DC, Mosteller, F., Tukey, J. W. Understanging Robust and Exploratory Data Analysis. Wiley Classics Library edition. 1982:472.
